# *Pten* Regulates Epithelial Cytodifferentiation during Prostate Development

**DOI:** 10.1371/journal.pone.0129470

**Published:** 2015-06-15

**Authors:** Isabel B. Lokody, Jeffrey C. Francis, Jennifer R. Gardiner, Janine T. Erler, Amanda Swain

**Affiliations:** 1 Division of Cancer Biology, The Institute of Cancer Research, 237 Fulham Road, London, United Kingdom; 2 Biotech Research and Innovation Centre, University of Copenhagen, Copenhagen, Denmark; Thomas Jefferson University, UNITED STATES

## Abstract

Gene expression and functional studies have indicated that the molecular programmes involved in prostate development are also active in prostate cancer. *PTEN* has been implicated in human prostate cancer and is frequently mutated in this disease. Here, using the *Nkx3*.*1*:*Cre* mouse strain and a genetic deletion approach, we investigate the role of *Pten* specifically in the developing mouse prostate epithelia. In contrast to its role in other developing organs, this gene is dispensable for the initial developmental processes such as budding and branching. However, as cytodifferentiation progresses, abnormal luminal cells fill the ductal lumens together with augmented epithelial proliferation. This phenotype resembles the hyperplasia seen in postnatal *Pten* deletion models that develop neoplasia at later stages. Consistent with this, gene expression analysis showed a number of genes affected that are shared with *Pten* mutant prostate cancer models, including a decrease in androgen receptor regulated genes. In depth analysis of the phenotype of these mice during development revealed that loss of *Pten* leads to the precocious differentiation of epithelial cells towards a luminal cell fate. This study provides novel insight into the role of *Pten* in prostate development as part of the process of coordinating the differentiation and proliferation of cell types in time and space to form a functional organ.

## Introduction

The mammalian prostate is a male specific structure that develops from the urogenital sinus under the influence of androgens [1]. In the mouse, androgen dependent signals from the mesenchyme induce the budding of the urogenital sinus epithelium, which become visible around 17.5 days of embryonic development (E17.5). The epithelial prostatic buds grow out into the surrounding mesenchyme and go through the processes of branching morphogenesis, canalization and cytodifferentiation into basal and luminal cells. These, together with rare neuroendocrine cells give rise to a fully functional adult organ. Gene expression studies have indicated that molecular programmes that are specific to prostate development are also active in prostate cancer [2,3]. Therefore analyses of pathways during organ development have provided important information as to the role of signalling molecules in prostate neoplasia.

PTEN (phosphatase and tensin homolog deleted on chromosome 10) tumour suppressor gene is part of the PI3K signalling pathway cascade and has been shown to be important in both organ development and in cancer. *Pten* deficient mice die in utero showing that *Pten* is essential for development [4]. The role of *Pten* has been specifically studied in the development of the lung, mammary gland and skin where it has been shown to regulate differentiation, branching and proliferation [5–7]. The role of *Pten* during prostate development has not been investigated to our knowledge.

PTEN has been implicated in human prostate cancer. Deletions and/or mutations in this gene are found in up to 30% of primary and 63% of metastatic prostate cancer samples [8–10]. Consistent with this, mice with a heterozygous mutation in *Pten* develop prostate intraepithelial neoplasia (PIN) after 10 months [11]. The creation of mice with a conditional allele of *Pten* (*Pten* ^*Fl/Fl*^) and mice with a construct that drives *Cre* recombinase expression in adult prostate epithelial cells (*PbCre4*) allowed the generation of mice with an adult prostate epithelia-specific deletion of *Pten* [12]. These mice develop PIN at 6 weeks of age and invasive carcinoma after 9 weeks. High levels of phosphorylated AKT were found associated with the neoplastic phenotype implicating the PI3K pathway in tumour development.

In this study we have used a *Cre* expressing strain of mice driven by the *Nkx3*.*1* promoter (*Nkx3*.*1*:*Cre* mice) to delete *Pten in vivo* during prostate development. In contrast to other prostate specific *Cre* strains which express *Cre* postnally, *Nkx3*.*1*:*Cre* is expressed in all epithelial cells from early stages of development into the adult [13]. Our data reveal that *Pten* is not required for the budding and branching stages of development. However, during cytodifferentiation, mutant animals have increased numbers of abnormally shaped luminal cells, which leads to cell filled lumens and an increase in prostate wet weights. In depth analysis of various prostate development markers show that the absence of PTEN leads to an acceleration of luminal cell differentiation associated with an increase in proliferation and an inhibition of androgen receptor (AR) dependent pathways.

## Materials and Methods

### Mouse breeding and strains

All mouse work was carried out in accordance with the Institute of Cancer Research (ICR) guidelines and with the UK Animals (Scientific Procedures) Act 1986 and approved by the ICR Animal Welfare and Ethical Review Body. The *Nkx3*.*1*:*Cre* line was obtained from Michael Shen and the *Pten* ^*Fl/Fl*^ line was obtained from Jackson Laboratories. The *Nkx3*.*1*:*Cre* allele was produced by inserting the *Cre* gene into the *Nkx3*.*1* locus by homologous recombination [13]. The *Pten* ^*Fl/Fl*^ allele was created by inserting *LoxP* sites flanking exon 5 of the *Pten* gene [14]. Mice were bred on a mixed genetic background according to standard protocols. The day of birth was designated post-natal day 0 (P0). All mutant *Nkx3*.*1*:*Cre; Pten* ^*Fl/Fl*^ animals in this study were heterozygous for the Nkx3.1:Cre allele. Control animals are defined as those without the Nkx3.1:Cre allele. Mice were sacrificed by the Schedule 1 methods of cervical dislocation or decapitation according to the UK Animals Act 1986.

### β-galactosidase staining

Following dissection in PBS, tissues were fixed for 1 hour at 4°C in 4% paraformaldehyde (PFA) and then washed several times in PBS. Subsequently tissue was stained with *β*-galactosidase solution (1 mg/ml Xgal, 5 mM K3Fe(CN)6, 5 mM K4Fe(CN)6, 2 mM MgCl2 and 0.02% NP40) at 37°C for 2 hours in the absence of light.

### Immunohistochemistry

The following antibodies and their respective concentrations were used for immunohistochemistry: PTEN (Cell Signalling, 9559) at 1:100 dilution, pAKT (Ser 473, Cell Signalling, 3787S) at 1:100 dilution, CK18 (Progen, 61028) at 1:2 dilution, p63 (Santa Cruz, SC-8431) at 1:200 dilution, CK5 (Covance, PRB-160P) at 1:50 dilution, CK8 (Covance, MMS-162P) at 1:50 dilution, Ki67 (DAKO, M7249, Clone TEC-3) at 1:200 dilution, TROP-2 (R&D systems, AF1122) at 1:50 dilution, PBSN (Santa Cruz Biotechnology, SC17124) at 1:200 dilution, CLU (Abcam, AB23375) at 1:400 dilution, αSMA (Sigma, #A2547) at 1:4000 dilution, Vimentin (Abcam, AB92547) at 1:1000 dilution, Synaptophysin (Dako, M0776) at 1:50 dilution, Androgen Receptor (Millipore, 06–680) at 1:250 dilution. Antibody staining was done on sections of paraffin-embedded tissue using standard protocols [15]. For DAB chromogen staining the ABC vector kit was used with biotinlyated secondary antibodies (Vector Laboratories) according to manufacturer's instructions and the DAB substrate (Dako). Secondary fluorescent antibodies were obtained from Molecular Probes and were used at a 1:500 dilution. For the Ki67/p63 and CK18 immunofluorence staining the Tyramide Signalling Amplification (TSA) Kit (PerkinElmer) was used. The CK18 or Ki67 TSA stain was carried out following the manufacturers instructions, followed by p63 stain using a Molecular Probes secondary antibody.

### Whole mount In situ hybridization

The *in situ* hybridization probes for *Nkx3*.*1* were generated from linearised DNA constructs containing T7 RNA polymerase recognition sites and have been previously described [13]. An RNA Labelling Kit (Roche) was used according to the manufacturer’s instructions to produce digoxigenin-labelled antisense riboprobes. Riboprobes were purified using Chromaspin 100 columns (Clontech). *In situ* hybridisation (ISH) was performed according to standard methods [16].

### Microarray

Total RNA was extracted from frozen P10 anterior and ventral prostates using an RNeasy Micro kit (Qiagen) according to the manufacturer’s instructions. 5 samples of each genotype (*Nkx3*.*1*:*Cre; Pten* ^*Fl/Fl*^ and control) were used. Microarray analysis (affymetrix based GeneChip Mouse Gene 1.0 ST Array) was carried out at the Paterson Institute Microarray Service (CRUK, Manchester). All data analysis was carried out at the Transcript Cluster ID level. DABG (detection above background) calling was done using Affymetrix Power Tools software. Individual CEL files were read into R using the Simpleaffy package and normalized using RMA. Cluster IDs were filtered according to DABG. The threshold used was a p value of < 0.01. If the p value was less than the threshold, the ID was present, if above the threshold, the ID was called absent. IDs were retained if they were called present in all samples, if they were called present in all samples in the control group and if they were called present in all samples in the mutant group. LIMMA was used to define differentially expressed transcript clusters, at a threshold of log2 fold change of 1 (linear fold change of 2).

### Gene network analysis

Network studies were performed by analysing the genes differentially expressed in the *Pten* mutant prostate compared to the control prostate using the Database for Annotation, Visualization and Integrated Discovery (DAVID, v6.7) bioinformatics functional annotation tool. Gene function was assigned using the Biological Process Gene Ontology data.

### Quantitative RTPCR

Total RNA was extracted from frozen tissue using the RNAeasy Micro Kit (Qiagen) as used for the microarray analysis. cDNA was synthesized with random primers using Superscript II reverse transcriptase (Invitrogen) and amplified with specific primers sequences (S1 Table). Quantitative RTPCR was carried out using SyBr Green (Invitrogen). The relative mRNA accumulation was determined using the ΔΔCt method with GAPDH for normalization. For statistical analysis, the unpaired t-test was used and P<0.05 was considered to be statistically significant.

### Quantitation of prostate wet weight and cell proliferation

The wet weight of control and mutant prostate was determined by measuring the mass of the urogenital sinus plus all prostatic lobes without seminal vesicle or bladder tissue. Ki67 staining was performed on prostate sections from 5 animals. Sections were also stained with p63 to define the borders of the ducts, and counterstained with DAPI to count the total number of cells in the ducts. The proliferation rate, that is, the number of proliferating cells per duct, was counted in each section and compared. An unpaired student’s t-test was used to determine statistical significant difference, with a p value of less that 0.05.

## Results

### *Pten* is not required for the early stages of prostate development

**Fig 1:**
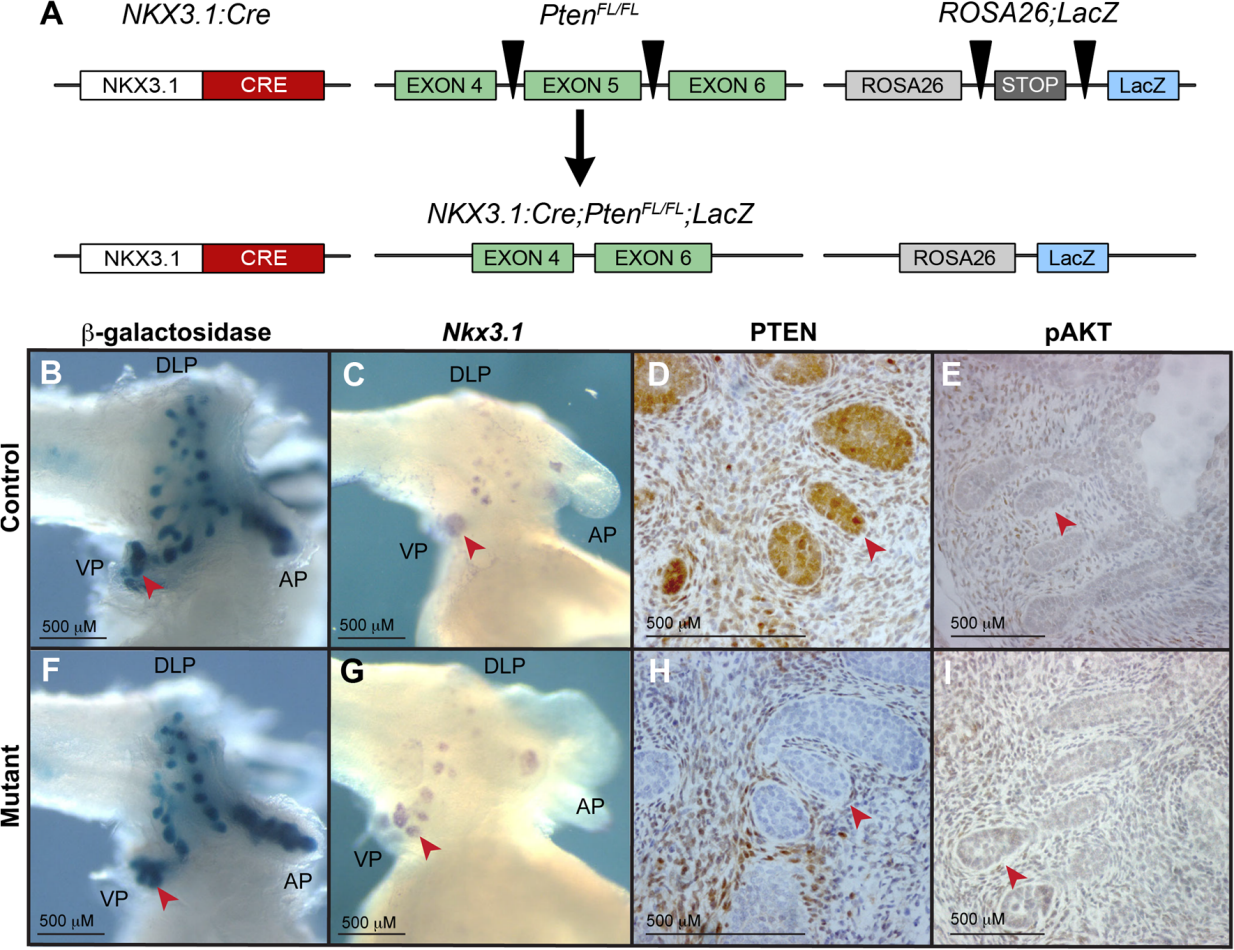
*Nkx3*.*1*:*Cre; Pten* ^*Fl/Fl*^ prostates appear normal at P0. (A) A gene diagram of the alleles used to generate the *Pten* mutant animals with *R26R* reporter allele. R26R is Rosa26 Cre reporter strain. Black triangles denote the position of LoxP sites. Morphological changes such as budding are unaffected in mutant (F) compared to control (B) prostates as exhibited by β galactosidase staining. Whole mount *in situ* hybridization of *Nkx3*.*1* shows expression in the mutant (G) similar to the control (C). Antibody staining on sections of P0 prostates demonstrates that PTEN is absent from epithelial buds in mutant (H) compared to control (D), and pAKT is not upregulated in mutant (I) relative to the control (E). Red arrows indicate prostatic buds, scale bars are as indicated. VP indicates ventral prostate, AP indicates anterior prostate and DLP indicates dorsal-lateral prostate.

We bred the *Nkx3*.*1*:*Cre* strain of mice to the *Pten* conditional allele containing mice to generate *Nkx3*.*1*:*Cre;Pten* ^*Fl/Fl*^ animals (hereafter called mutant). Previous work has established that the *Nkx3*.*1*:*Cre* strain displays Cre recombinase activity in the prostatic epithelial buds from E17.5, making it an ideal model to study gene function during prostate development [13]. In order to highlight morphological changes in budding and branching in the developing prostate, mutant mice were bred with the *ROSA26* derived *Cre* reporter strain *R26R* [17] (Fig 1A). In this reporter strain, tissues that contain active Cre protein express *LacZ*. Whole mount analysis of P0 prostates derived from this breeding showed no major changes in prostate budding and branching between the control and mutant animals carrying the *R26R* allele (Fig 1B and 1F). Consistent with this, whole mount *in situ* hybridisation studies showed that *Nkx3*.*1* expression, which specifically marks the early prostate epithelia, was not altered in the mutant organ at P0 (Fig 1C and 1G). To investigate the extent of *Pten* deletion in mutant animals, we analysed the prostate from P0 animals using an antibody to PTEN. These studies showed an absence of the protein throughout the mutant epithelia, demonstrating *Pten* deletion in all bud cells, as expected (Fig 1D and 1H). Accumulation of phosphorylated AKT is a feature of PTEN deletion in many tissues [18], therefore we also stained P0 prostatic buds with a phospho-AKT (pAKT) specific antibody. Surprisingly we saw no major accumulation in the mutant prostate at this stage (Fig 1E and 1I).

### *Pten* loss disrupts prostate lumen formation and proper cytodifferentiation

**Fig 2:**
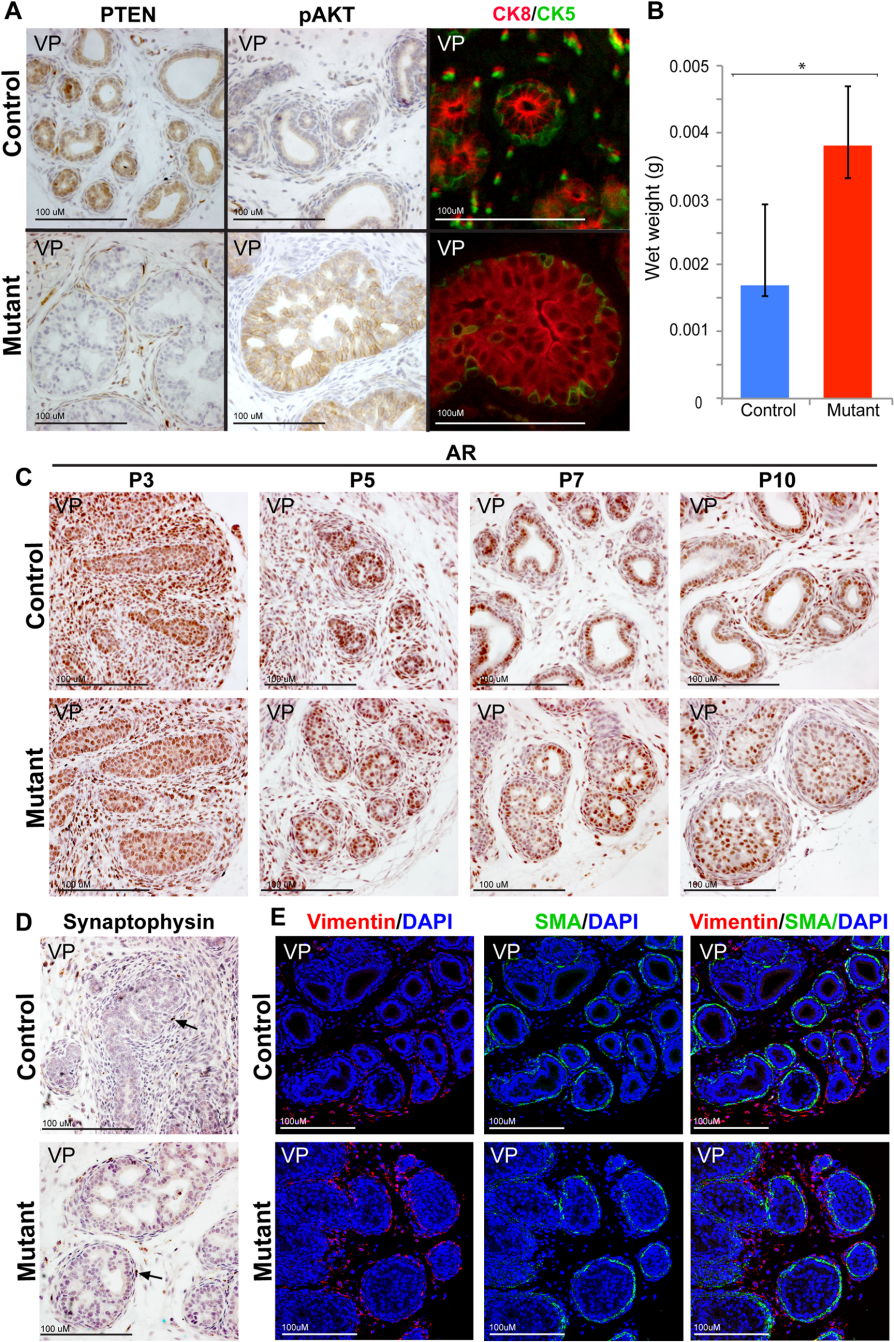
*Nkx3*.*1*:*Cre;Pten* ^*Fl/Fl*^ mice exhibit a cellular phenotype at P10. (A) Antibody staining of P10 prostate sections demonstrates that mutant mice exhibit loss of PTEN, increased expression of pAKT, of organization of CK8 (red) luminal cells that fill the duct and CK5 (green) basal cells. (B) P10 mutant mice exhibit a statistically significant (*, p = 0.002, n = 10) increase in prostate wet weight compared to control mice. Antibody staining on sections of prostates demonstrates that (C) AR expression is similar in the mutant compared to controls at P3, P5, P7 and P10, (D) rare neuroendocrine cells marked by Synaptophysin are present at P10 in the mutant and controls (black arrow), and (E) Vimentin and Smooth Muscle Actin (SMA) expression shows that mesenchymal differentiation has taken place at P10 in the mutant similar to controls. Scale bars are as indicated. All sections are ventral prostate (VP).

Histological analysis of animals at 10 days of postnatal development (P10) revealed an abnormal phenotype in the mutant *Pten* prostate. At this stage, epithelial cells in the normal prostate have differentiated into basal and luminal cells. Antibody staining for PTEN confirmed the absence of this protein in both cell types in the mutant tissue (Fig 2A). In contrast to P0, a high level of pAKT was observed in the mutant P10 prostate (Fig 2A). Staining with antibodies to a luminal marker, CK8 (cytokeratin 8), and a basal marker, CK5 (cytokeratin 5), revealed that the structure of the prostatic ducts was affected in the mutant prostate (Fig 2A). There was a marked increase in CK8 positive cells that filled the ducts disturbing normal lumen formation. In addition, the classic morphology of both luminal and basal cells was affected in the mutant animals. Most mutant luminal cells had failed to differentiate into the columnar shape of epithelial cells present in control prostates and were more polygonal. Furthermore, mutant basal cells did not show a classic triangular morphology (Fig 2A). This cellular phenotype has similar characteristics to that found in studies where *Pten* was deleted using a Cre expressing mouse line driven by the *PSA* promoter. The *PSA-Cre* line leads to mosaic gene deletion in luminal cells at late postnatal stages [19,20]. These animals display abnormal luminal structure and cellular differentiation before the appearance of PIN. Consistent with the expansion in the number of luminal cells, a significant increase in prostate wet weight was observed at P10 between mutant and control animals (Student t-test p = 0.002) (Fig 2B).

Further analysis of prostate cell type markers was performed on mutant and control prostates at various developmental stages. Immunohistochemical studies showed that AR expression was not substantially altered in the epithelia or mesenchyme of P3, P5, P7 or P10 prostates (Fig 2C). Antibody staining for Vimentin and Smooth Muscle Actin in mutant and control prostates showed that mesenchymal differentiation was normal in *Pten* deleted mice (Fig 2E). Rare neuroendocrine cells, marked by Synaptophysin, were present in control and mutant prostates, indicating that *Pten* deletion in prostate epithelia during development does not affect the number of this cell type (Fig 2D).

**Fig 3:**
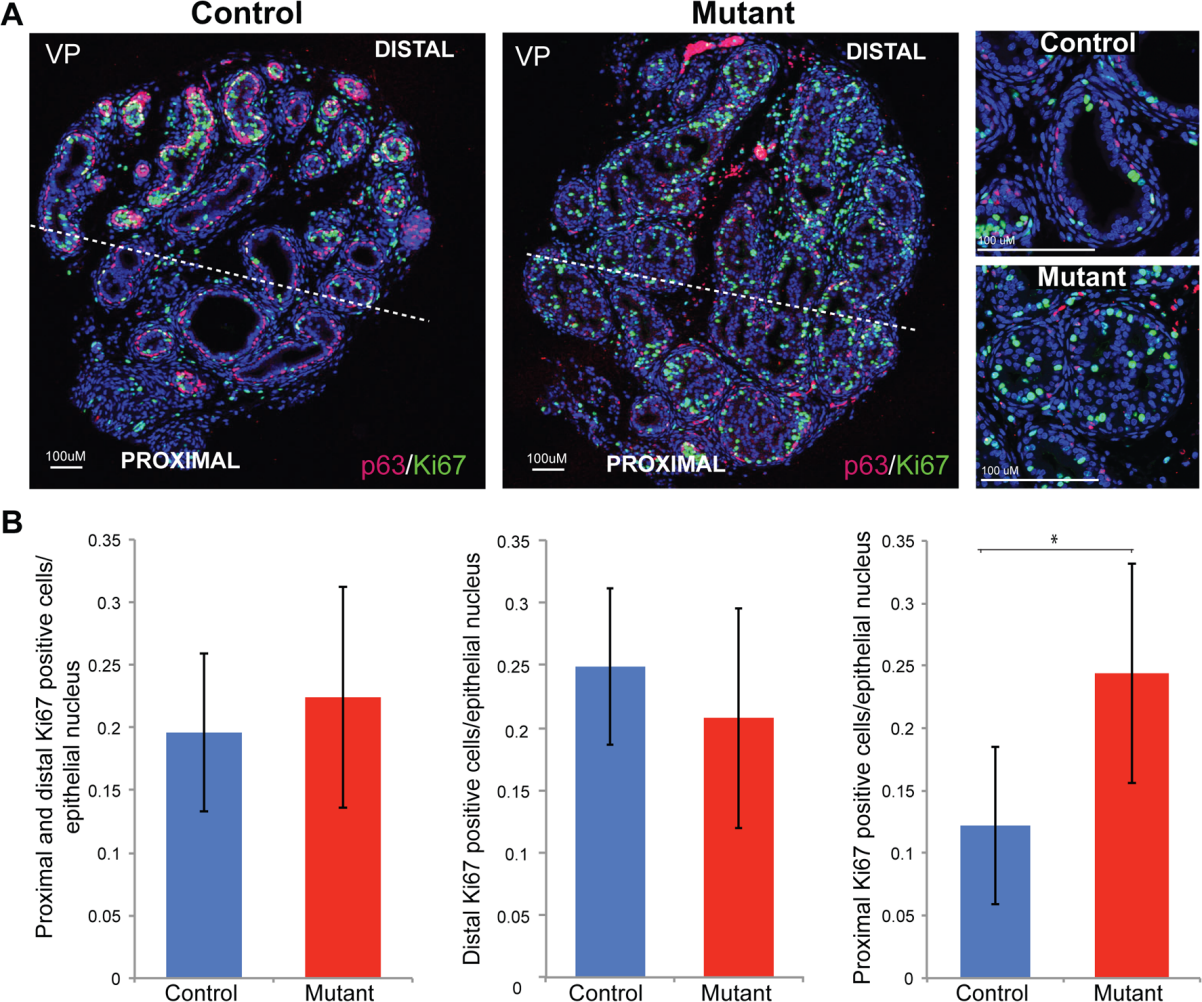
*Pten* mutant prostates have an increase in proximal proliferation. (A) Double immunofluorescence antibody staining of P10 mutant and control sections with Ki67 (green) and p63 (red) antibodies demonstrate a loss in proximal-distal patterning in mutant prostates. Right panels, high magnification of proliferating Ki67 expressing cells and basal p63 expressing cells (B) Left panel, quantification of Ki67 positive cells in control and mutant prostates. Middle panel, quantification of Ki67 positive cells in the distal area only shows no statistically significant difference. Right panel, quantification of Ki67 positive cells in the proximal area only shows a statistically significant increase (*, p = 0.04, n = 5). The proximal to distal boundary is indicated by the dashed line in A; error bars are standard deviations. VP indicates ventral prostate.

Analysis of Ki67 expression, which marks proliferating cells, showed a change in the proximal-distal pattern in the mutant compared to that observed in controls. During normal prostate development, cells at the tips of the prostatic buds (distal to the developing urethra) display high levels of proliferation in comparison to the intermediate or more proximal cells [21]. This pattern was absent in *Pten* mutant animals, which showed high levels of Ki67 throughout the prostate (Fig 3A). Quantitation studies showed a statistically significant difference in the number of proliferating cells between control and mutant when the proximal (Student’s t-test p = 0.04), but not the distal (Student’s t-test p = 0.51), region of the prostate was compared (Fig 3B).

### Expression of AR regulated genes is affected by *Pten* loss

**Fig 4:**
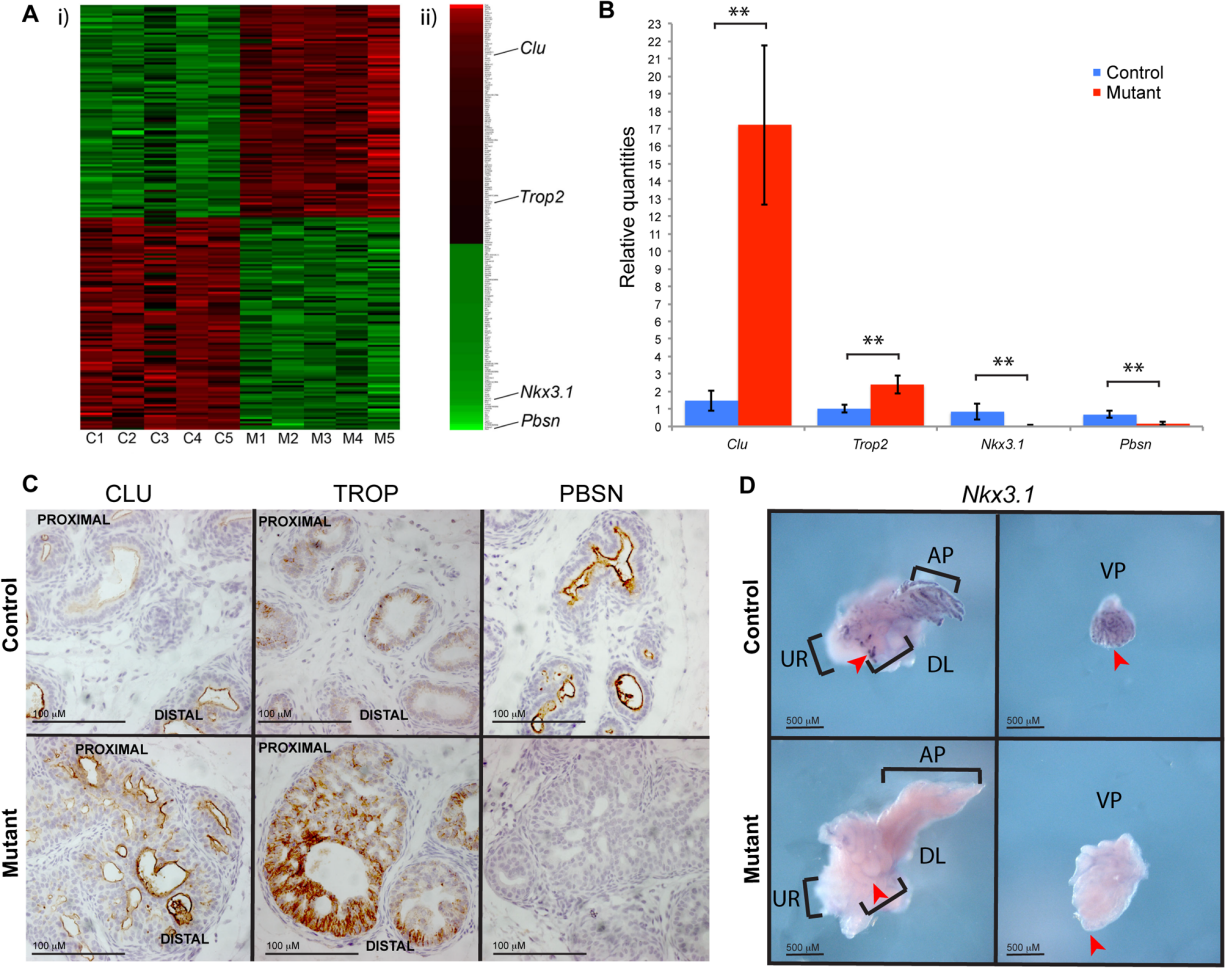
Gene expression analysis of *Pten* mutant prostates. (A) i) Heatmap demonstrating that control samples (C1-C5) show similar expression patterns to each other, as do mutant samples (M1-M5). ii) Genes chosen for validation are indicated in heatmap derived from the average of control and mutant samples. Red indicates genes that are upregulated in mutants (116 genes), relative to controls, and green indicates genes that are downregulated in the mutants relative to controls (91 genes). (B) Quantitative RTPCR validation of indicated androgen-dependent genes differentially expressed in mutants and controls (*Clu* p = 0.001, *Trop2* p = 0.002, *Nkx3*.*1* p = 0.02, and *Pbsn* p = 0.003). mRNA accumulation was normalized to GAPDH. (C) Differential antibody staining of CLU, TROP2, and PBSN at P10 validated microarray results. (D) Whole mount in situ hybridization of *Nkx3*.*1* shows a decrease in expression in P10 mutant prostates. The anterior prostate (AP), dorsal lateral prostate (DL) and urethra (UR) are indicated. Red arrows indicate presence of stain in the controls and absence in the mutant. “*” denotes statistical significance.

To investigate the pathways that are affected by *Pten* deletion in the developing prostate we performed a differential expression microarray analysis. RNA was extracted from prostatic tissue from P10 control and mutant animals and expression profiles were compared. Statistical analysis was used to generate a list of genes that were either significantly upregulated (116 genes) or downregulated (91 genes) in the mutant compared to control animals (Fig 4A and list of genes shown in S2 Table). Consistent with similar aspects of the luminal cell phenotype, several candidates were shared with other expression studies using mice expressing *Cre* driven by *Probasin* or *PSA* derived promoters to delete *Pten* in the adult prostate [12,19,22,23]. In particular, we observed a change in expression of several proposed androgen dependent genes including *Probasin* (*Pbsn*) and *Nkx3*.*1*, which are downregulated in mutant prostates, and *Tacstd2* (*Trop2*) and *Clusterin* (*Clu)*, which are upregulated (see S3 and S4 Tables for a list of genes that have been proposed to be androgen regulated). Validation of the array results was performed on tissue from P10 prostates by quantitative RTPCR (Fig 4B) and by antibody staining for CLU, TROP2 and PBSN (Fig 4C). *Nkx3*.*1* expression was investigated using whole mount *in situ* hybridisation, which showed a severe decrease in signal in the mutant compared to the control (Fig 4D). This decrease was also seen when compared to P10 prostates from *Nkx3*.*1*:*Cre;Pten* ^*Fl/+*^ animals, which are also heterozygous for the *Nkx3*.*1* allele (S1 Fig). Network analysis was performed using the list of genes differentially expressed between *Pten* mutant and wild type animals (S5 Table). This study revealed a significant association with genes involved in lipid and steroid, including cholesterol biosynthesis.

### *Pten* deletion does not block prostate cytodifferentiation

**Fig 5:**
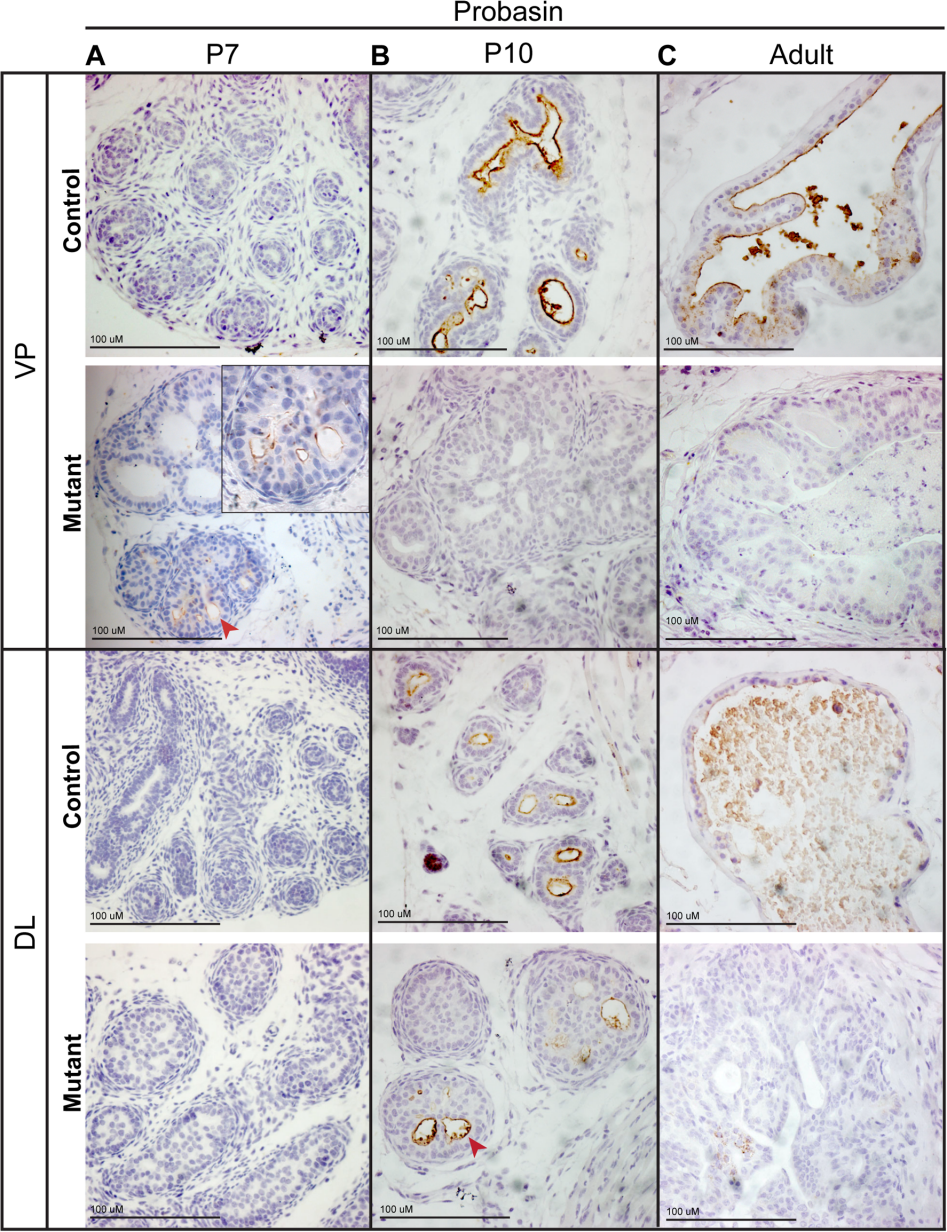
*Pten* deletion does not lead to a block in terminal differentiation. Antibody staining on sections of P7 (A), P10 (B) and adult (C) prostates demonstrate expression of PBSN in the mutant at early stages followed by loss of expression at later stages. PBSN expression is constant in the controls after P10. Peak PBSN expression in mutants varies by lobe. VP ventral prostate; DL dorsal lateral prostate. Red arrowheads indicate ducts.

Prostate epithelial cell differentiation follows a series of defined steps. Initially, the prostate is composed of compact epithelial cords with non-polarized cells that express p63. As canalization proceeds, cells become polarized with respect to the basal lamina and lumens appear. At the same time, the epithelial cells differentiate into the two main cell types, the luminal cells, marked by cytokeratins 8 and 18 (CK8, CK18) and loss of p63, and the basal cells, marked by CK5 and maintenance of p63 expression [24,25]. Final stages of epithelial differentiation include the appearance of secretory function by luminal cells, which is regulated, in part, by the epithelial androgen receptor (AR) [26]. Our expression studies suggest that *Pten* deletion leads to deregulation of epithelial AR function, highlighted by the downregulation of the secretory androgen regulated protein PBSN in the mutant animals. To investigate if *Pten* is required for the final stages of luminal cell differentiation, we performed PBSN antibody staining at different stages of development on prostates from control and mutant samples. Our results show that PBSN can be seen in the mutant prostate but this expression is extinguished at later stages. The stages of peak PBSN expression were found to be specific for each lobe, with the ventral lobe showing staining at P7 but not P10 and the dorsal lobe at P10 but not at later stages (Fig 5). This data suggest that prostate luminal cell differentiation does occur in the absence of *Pten*.

### TROP2 and CLU do not mark undifferentiated prostate epithelial cells

**Fig 6:**
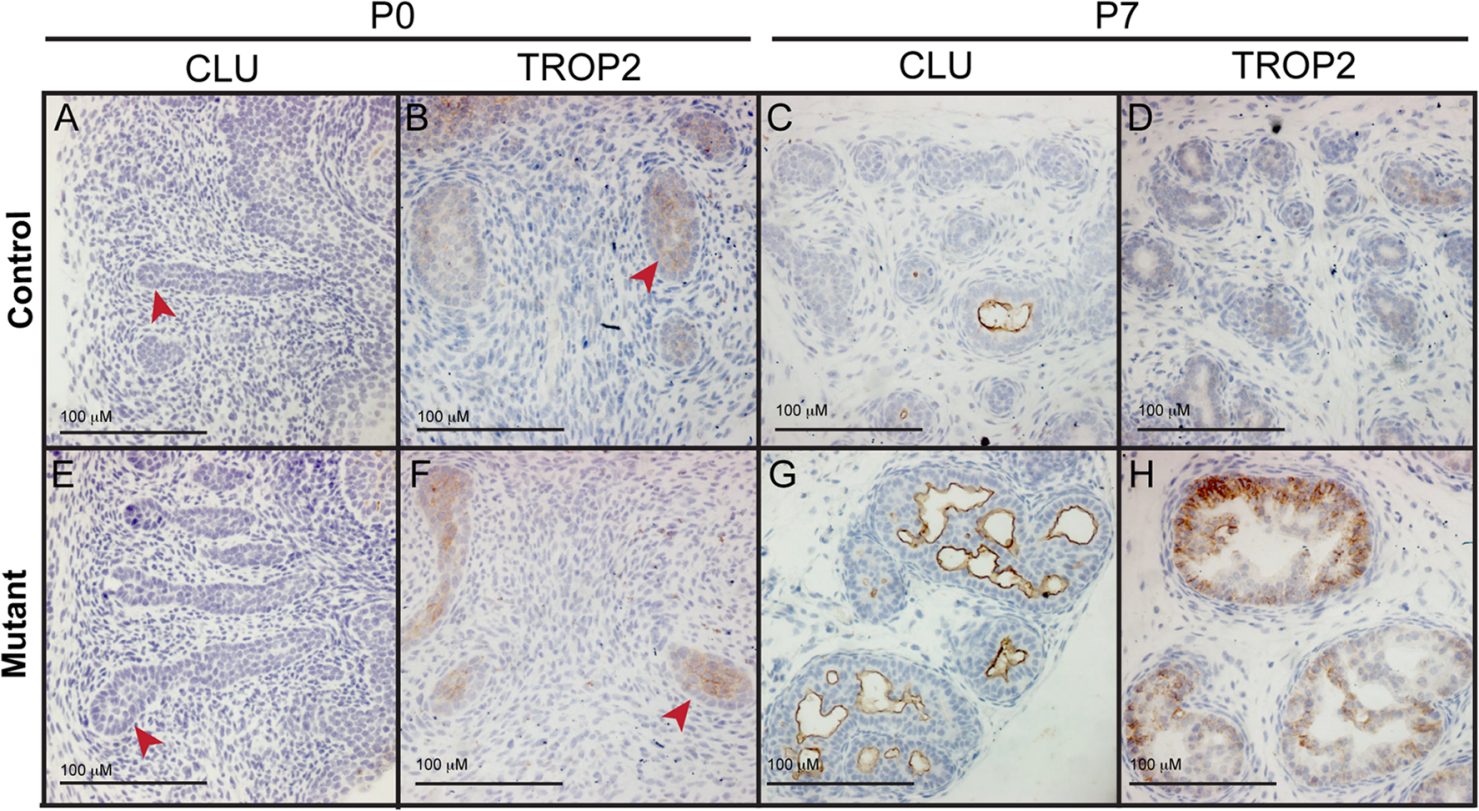
*Pten* deletion does not lead to an accumulation of progenitor cells. Antibody staining on sections of P0 and P7 prostates with antibodies to CLU and TROP2 shows no accumulation of these proteins at earlier stages of development in controls (A, B, C, D). Increased expression of these markers is seen in the mutant (E, F, G, H) relative to controls from P7. Red arrowheads indicate buds.

Several studies on mice with deletion of *Pten* in adult prostate epithelial cells have suggested that lack of this gene leads to an accumulation of progenitor cells, which are normally found in regions proximal to the urethra in control adult animals [27,28]. Two markers, TROP2 and CLU, have been proposed to mark these progenitor cells in control animals [19,27]. CLU is a glycoprotein molecular chaperone implicated in many cellular processes, including apoptotic pathways. CLU can be induced by stress, such as anti-cancer therapies, and drugs targeting this protein are being tested as therapeutics in prostate cancer patients [29,30]. TROP2 is a cell surface receptor that transduces calcium signals. *TROP2* is overexpressed in a number of cancer cell lines, including prostate, and can regulate self-renewal, proliferation, transformation and migration [31,32]. Our data show that these markers are upregulated in the mutant prostate at P10. Therefore we analysed the expression of TROP2 and CLU at earlier postnatal stages in control and mutant animals to investigate whether they revealed a similar phenotype to that of the adult *Pten* deleted mice. Our results show that these markers do not accumulate in the early (P0 and P7) control prostate (Fig 6). In the mutant prostate, increased levels of both TROP2 and CLU in luminal cells are seen as early as P7 and are maintained throughout development. In addition we do not observe any proximal-distal expression patterning of these markers in the control and mutant prostate at the stages analysed (Fig 6). These data show that TROP2 and CLU do not mark early, undifferentiated cells during normal prostate development. Our studies suggest that the increase in expression of these markers in the mutant prostate does not reflect undifferentiated postnatal epithelial cells and is an independent process to that of adult cell precursor formation.

### *Pten* mutant prostates exhibit precocious luminal cell differentiation

**Fig 7:**
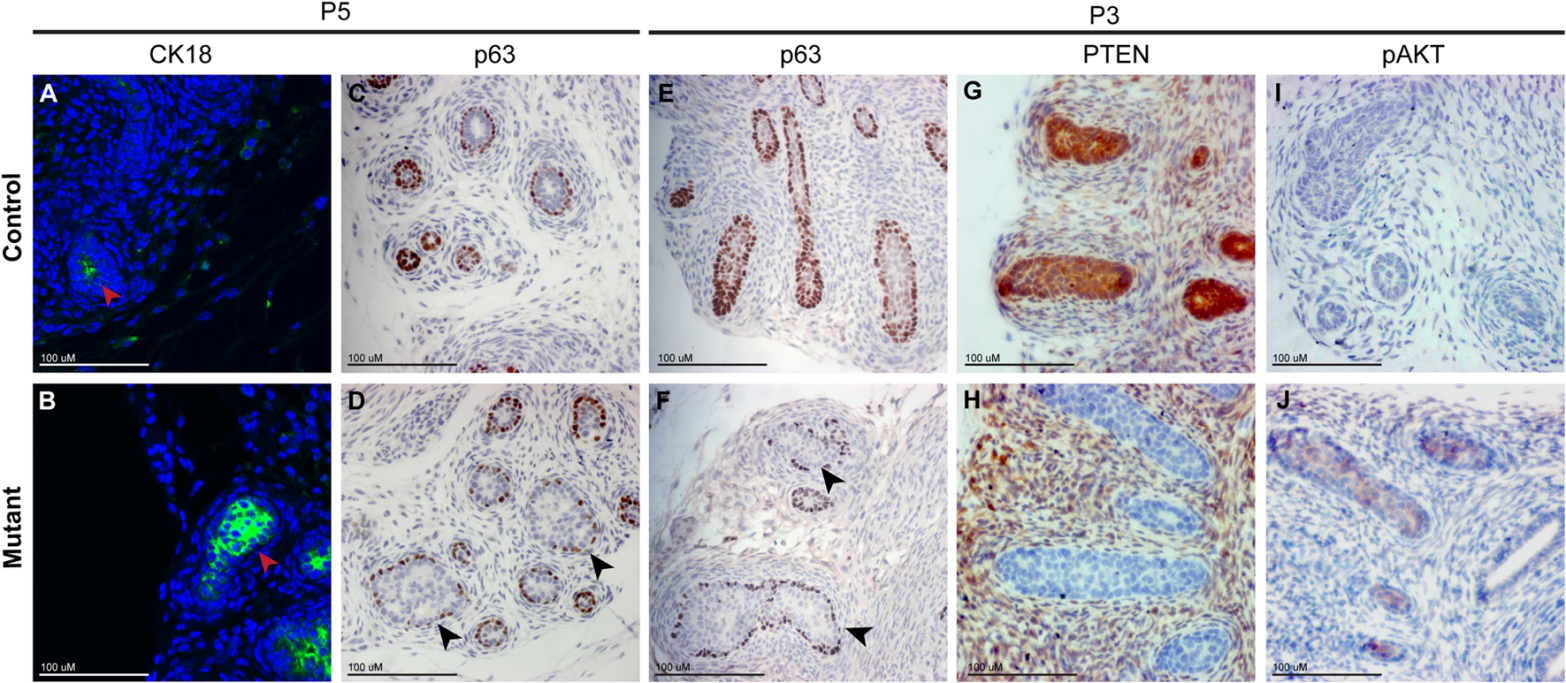
*Pten* mutant prostates exhibit precious cytodifferentiation. Antibody staining of the luminal marker CK18 (green) on sections of P5 prostates show expression is increased mutant (B) prostatic ducts compared to controls (A). Red arrowheads indicate ducts. Antibody staining of the basal cell marker p63 on sections of P5 and P3 prostates shows expression is downregulated in the mutant ducts (D,F) compared to controls (C,E), indicated with black arrowheads. Antibody staining for pAKT and PTEN on sections of P3 prostates show a loss of PTEN (H) and an upregulation of pAKT (J) in mutant buds compared to controls (G,I).

Our antibody analysis of P10 animals showed an increase in CK8 expression in the mutant prostate suggesting that luminal cell differentiation was more advanced in these mice (S2 Fig). This was consistent with the early expression of PBSN in the mutant compared to the control animal (see ventral prostate staining at P7 in Fig 5). Therefore we analysed the expression of CK8/CK18 at different stages of development to determine when luminal cells first differentiate in the mutant and control animals. Analysis at various stages showed that at 5 days of postnatal development (P5), cells within the prostatic ducts in the control animals showed very low levels of CK18. In contrast, in the mutant prostate expression of CK18 was clearly visible (Fig 7A and 7B). Luminal cell differentiation is also characterised by the downregulation of p63 expression, while this protein is retained in differentiating basal cells. Analysis of prostates from P5 and postnatal day 3 (P3) animals with an antibody to p63 shows mutant buds with an increase in cells that have downregulated p63 compared to control animals (Fig 7C–7F). At P3, PTEN is lost throughout mutant epithelial buds and pAKT has begun to accumulate (Fig 7G–7J). Therefore these data show that luminal cell differentiation is accelerated in *Pten* mutant prostates at a time when pAKT accumulates.

## Discussion

The importance of *Pten* loss in prostate cancer has highlighted the need to understand the role of this gene in the normal prostate. We have used a *Cre* expressing strain of mice that deletes *Pten* in the majority of prostate epithelia cells as they arise during embryogenesis. This is in contrast to other studies using *Cre* expressing lines such as the *PSA-Cre* and *PbCre4*, where *Pten* is deleted in a subset of prostate epithelial cells starting after postnatal stages and mostly found in luminal cells. Our studies show that this gene is not required for the early stages of prostate development. *Pten* mutant animals displayed epithelial cell differentiation into luminal and basal cells, however, an increase in luminal cells of abnormal shape that filled the lumens of the ducts was observed. In-depth analysis of the phenotype showed that loss of *Pten* led to acceleration in luminal cell differentiation, an increase in proliferation and deregulation of AR dependent pathways.

Published studies have shown that *Nkx3*.*1* haploinsufficiency can cause a mild phenotype in the adult prostate and that there is an additive effect with the loss of *Pten [33,34]*. Although our *Nkx3*.*1*:*Cre* line is effectively heterozygous for *Nkx3*.*1*, we did not observe a developmental phenotype, including *Nkx3*.*1* expression and pAKT staining, in our analysis of prostates from *Nkx3*.*1*:*Cre;Pten* ^*Fl/+*^ mice. Therefore we do not think that loss of an *Nkx3*.*1* allele has a major impact on the mutant prostate phenotype described here.

PTEN is a key regulator of the PI3K signalling pathway and loss of PTEN frequently leads to an accumulation of pAKT [18]. Our studies show an increase in pAKT in prostate epithelia at stages when a phenotype is observed in mutant animals. This implies that PI3K signalling through pAKT promotes luminal cell differentiation and proliferation and that the role of PTEN in the developing prostate is to restrict this function. The localisation of pAKT in mutant animals is mostly confined to the cell membrane and cytoplasm. Studies in human prostate cancer have shown that this site is associated with low grade PIN, whereas high grade tumours show nuclear pAKT [35].

Our data also demonstrate that PTEN is dispensable for early epithelial prostate developmental processes such as budding and branching. This is surprising as *Pten* deletion has profound consequences on early developmental processes in other organs such the developing mammary gland, where it causes excessive branching and precocious budding, which we did not observe in the prostate [5]. In addition, PI3K signalling has been shown to be important during prostate budding and branching morphogenesis, which we have confirmed using pharmacological inhibitors of PI3K in *ex vivo* organ cultures [36] (data not shown). However, at P0 we do not observe an accumulation of pAKT or a prostate phenotype in *Pten* deleted animals. This suggests that, at this stage, *Pten* does not regulate PI3K signalling and that this pathway is controlled through a different mechanism.

AR has been shown to be important at various stages of prostate development in both the epithelia and mesenchyme compartment [1]. During cytodifferentiation, upregulation of AR dependent secretory function marks the final stages of luminal cell development. Our studies show that loss of *Pten* initially leads to the acceleration of luminal cell differentiation, including the premature expression of AR dependent secretory markers, such as PBSN, but these are then downregulated. These data suggest that increased PI3K signalling interferes with the transcriptional activity of AR but does not prevent luminal cell differentiation taking place. Network analysis suggested that *Pten* deletion led to an increase in lipid and steroid biosynthesis. Interestingly, recent work has shown an increase in esterified cholesterol in prostate cancer samples, which were found to be dependent on *PTEN* loss [37]. However, this increase was shown to be due to an enhanced uptake of exogenous lipoproteins, whereas we see an increase in expression of enzymes involved in de novo cholesterol synthesis.

Studies in the adult *Pten* deleted mouse prostate have shown interplay between NKX3.1 and PTEN, in particular that loss of *Pten* leads to downregulation of Nkx3.1 expression [38]. Our data show that *Nkx3*.*1* expression is only affected at later postnatal stages in the *Pten* mutant animals. Our results are consistent with the hypothesis that the transcriptional regulation of *Nkx3*.*1* expression in the undifferentiated prostate epithelia at early stages of development is distinct from its expression in differentiated luminal cells, where it has been shown to be AR dependent. Therefore our data suggests that the repression of *Nkx3*.*1* by *Pten* loss in luminal cells is through the AR pathway.

Previous studies proposed that lack of *Pten* led to a more precursor-like phenotype in the prostate that promoted tumourigenesis [19,39]. We found no evidence to support this proposal. In contrast in our model, loss of *Pten* led to an acceleration of luminal cell differentiation with the increase of markers that did not represent early undifferentiated stages of prostate development. We did not observe the compartmentalization of proximal-distal regions seen in the normal adult prostate in the postnatal stages of control animals we analysed (up to P15). Therefore the process of proximal organization must occur at later stages and is disconnected to the appearance of the phenotype in our *Pten* mutant animals.

*Pten* loss and upregulation of the PI3K signalling pathway is well known to regulate proliferation. Despite an increase in wet weight, analysis of total Ki67 positive cells did not reveal a statistically significant increase in the number of proliferating cells in mutants. However, in mutant prostates there was a change in the proximal-distal localization of Ki67 positive cells, which in control prostates is primarily restricted to the tips of the buds. In mutants, this patterning is lost and high levels of Ki67 positive cells are seen throughout the prostate. This demonstrates that in the developing prostate *Pten* restricts the levels of proliferating cells to the outgrowing ductal tips.

Recent studies using lineage-specific *Pten* deletion in adult prostate cancer models have shown that basal cells are resistant to direct transformation and require transition into luminal cells to become neoplastic [40]. However, during normal adult prostate homeostasis basal and luminal cells are primarily generated from within their own lineage, both with rare bipotent cells [40–42]. This suggests that *Pten* loss in adult prostate basal cells causes deregulation of the differentiation program that leads to transition into luminal cells and promotes cancer initiation. This is consistent with our model, where *Pten* is deleted in all epithelial cells as the prostate forms, and results in an accumulation of luminal cells without an increase in basal cell number. In addition, we have shown that *Pten* loss promotes this accumulation by promoting the early differentiation towards a luminal cell phenotype. We have shown that this early differentiation towards a luminal cell type occurs when pAKT is upregulated, suggesting that PI3K dependent roles of *Pten* are initiating this differentiation process. These developmental studies highlight *Pten* as a key regulator of proliferation and differentiation of prostate epithelial cells. The investigation of *Pten* in prostate development gives further insight into the biology of *Pten* and its role in prostate cancer and other *Pten*-relevant malignancies.

## Supporting Information

S1 Fig Whole mount in situ hybridization for *Nkx3*.*1* shows a decrease in expression in P10 mutant prostates. *Cre-* controls and *Pten* heterozygotes (*Nkx3*.*1*:*Cre;Pten* ^*Fl/+*^) both express *Nkx3*.*1* in all lobes. The anterior prostate (AP), dorsal lateral prostate (DL) and urethra (UR) are indicated. Red arrows indicate presence of stain in the controls and absence in the mutant.
(TIF)

S2 Fig Double antibody staining for CK8 (cytoplasmic) and p63 (nuclear) on sections of P10 prostates. Mutant ducts are filled with luminal cells expressing high levels of cytoplasmic CK8. Red circles highlight basal cells that express nuclear p63.
(TIF)

S1 Table Primer sequences used for quantitative RTPCR. (XLSX)

S2 Table Differentially expressed genes from the *Nkx3*.*1*:*Cre;Pten* ^*Fl/Fl*^ microarray data. (XLSX)

S3 Table AR-responsive genes from Mulholland et al 2011. (XLSX)

S4 Table Potential AR regulated and Pten regulated genes from the Androgen Responsive Gene Database (ARGDB). (XLSX)

S5 Table Differentially expressed genes in Pten mutant prostate analysed using the DAVID bioinformatics functional annotation tool. (XLSX)

## References

[1] Cunha GR, Donjacour AA, Cooke PS, Mee S, Bigsby RM, Higgins SJ, et al The endocrinology and developmental biology of the prostate. Endocr Rev. 1987; 8: 338–362. 3308446 10.1210/edrv-8-3-338

[2] Schaeffer EM, Marchionni L, Huang Z, Simons B, Blackman A, Yu W, et al Androgen-induced programs for prostate epithelial growth and invasion arise in embryogenesis and are reactivated in cancer. Oncogene. 2008; 27: 7180–7191. 10.1038/onc.2008.327 18794802 PMC2676849

[3] Pritchard C, Mecham B, Dumpit R, Coleman I, Bhattacharjee M, Chen Q, et al Conserved gene expression programs integrate mammalian prostate development and tumorigenesis. Cancer Res. 2009; 69: 1739–1747. 10.1158/0008-5472.CAN-07-6817 19223557

[4] Di Cristofano A, Pesce B, Cordon-Cardo C, Pandolfi PP Pten is essential for embryonic development and tumour suppression. Nat Genet. 1998; 19: 348–355. 9697695 10.1038/1235

[5] Li G, Robinson GW, Lesche R, Martinez-Diaz H, Jiang Z, Rozengurt N, et al Conditional loss of PTEN leads to precocious development and neoplasia in the mammary gland. Development. 2002; 129: 4159–4170. 12163417 10.1242/dev.129.17.4159

[6] Suzuki A, Itami S, Ohishi M, Hamada K, Inoue T, Komazawa N, et al Keratinocyte-specific Pten deficiency results in epidermal hyperplasia, accelerated hair follicle morphogenesis and tumor formation. Cancer Res. 2003; 63: 674–681. 12566313

[7] Yanagi S, Kishimoto H, Kawahara K, Sasaki T, Sasaki M, Nishio M, et al Pten controls lung morphogenesis, bronchioalveolar stem cells, and onset of lung adenocarcinomas in mice. J Clin Invest. 2007; 117: 2929–2940. 17909629 10.1172/JCI31854 PMC1994617

[8] Dahia PL PTEN, a unique tumor suppressor gene. Endocr Relat Cancer. 2000; 7: 115–129. 10903528 10.1677/erc.0.0070115

[9] Suzuki H, Freije D, Nusskern DR, Okami K, Cairns P, Sidransky D, et al Interfocal heterogeneity of PTEN/MMAC1 gene alterations in multiple metastatic prostate cancer tissues. Cancer Res. 1998; 58: 204–209. 9443392

[10] Taylor BS, Schultz N, Hieronymus H, Gopalan A, Xiao Y, Carver BS, et al Integrative genomic profiling of human prostate cancer. Cancer Cell. 2010; 18: 11–22. 10.1016/j.ccr.2010.05.026 20579941 PMC3198787

[11] Podsypanina K, Ellenson LH, Nemes A, Gu J, Tamura M, Yamada KM, et al Mutation of Pten/Mmac1 in mice causes neoplasia in multiple organ systems. Proc Natl Acad Sci U S A. 1999; 96: 1563–1568. 9990064 10.1073/pnas.96.4.1563 PMC15517

[12] Wang S, Gao J, Lei Q, Rozengurt N, Pritchard C, Jiao J, et al Prostate-specific deletion of the murine Pten tumor suppressor gene leads to metastatic prostate cancer. Cancer Cell. 2003; 4: 209–221. 14522255 10.1016/s1535-6108(03)00215-0

[13] Thomsen MK, Butler CM, Shen MM, Swain A Sox9 is required for prostate development. Dev Biol. 2008; 316: 302–311. 10.1016/j.ydbio.2008.01.030 18325490

[14] Lesche R, Groszer M, Gao J, Wang Y, Messing A, Sun H, et al Cre/loxP-mediated inactivation of the murine Pten tumor suppressor gene. Genesis. 2002; 32: 148–149. 11857804 10.1002/gene.10036

[15] Francis JC, Thomsen MK, Taketo MM, Swain A beta-catenin is required for prostate development and cooperates with Pten loss to drive invasive carcinoma. PLoS Genet. 2013; 9: e1003180 10.1371/journal.pgen.1003180 23300485 PMC3536663

[16] Gardiner JR, Jackson AL, Gordon J, Lickert H, Manley NR, Basson MA Localised inhibition of FGF signalling in the third pharyngeal pouch is required for normal thymus and parathyroid organogenesis. Development. 2012; 139: 3456–3466. 10.1242/dev.079400 22912418 PMC3424047

[17] Soriano P Generalized lacZ expression with the ROSA26 Cre reporter strain. Nat Genet. 1999; 21: 70–71. 9916792 10.1038/5007

[18] Stambolic V, Suzuki A, de la Pompa JL, Brothers GM, Mirtsos C, Sasaki T, et al Negative regulation of PKB/Akt-dependent cell survival by the tumor suppressor PTEN. Cell. 1998; 95: 29–39. 9778245 10.1016/s0092-8674(00)81780-8

[19] Korsten H, Ziel-van der Made A, Ma X, van der Kwast T, Trapman J Accumulating progenitor cells in the luminal epithelial cell layer are candidate tumor initiating cells in a Pten knockout mouse prostate cancer model. PLoS One. 2009; 4: e5662 10.1371/journal.pone.0005662 19461893 PMC2680948

[20] Ma X, Ziel-van der Made AC, Autar B, van der Korput HA, Vermeij M, van Duijn P, et al Targeted biallelic inactivation of Pten in the mouse prostate leads to prostate cancer accompanied by increased epithelial cell proliferation but not by reduced apoptosis. Cancer Res. 2005; 65: 5730–5739. 15994948 10.1158/0008-5472.CAN-04-4519

[21] Sugimura Y, Cunha GR, Donjacour AA, Bigsby RM, Brody JR Whole-mount autoradiography study of DNA synthetic activity during postnatal development and androgen-induced regeneration in the mouse prostate. Biol Reprod. 1986; 34: 985–995. 3730490 10.1095/biolreprod34.5.985

[22] Mulholland DJ, Tran LM, Li Y, Cai H, Morim A, Wang S, et al Cell autonomous role of PTEN in regulating castration-resistant prostate cancer growth. Cancer Cell. 2011; 19: 792–804. 10.1016/j.ccr.2011.05.006 21620777 PMC3157296

[23] Carver BS, Chapinski C, Wongvipat J, Hieronymus H, Chen Y, Chandarlapaty S, et al Reciprocal feedback regulation of PI3K and androgen receptor signaling in PTEN-deficient prostate cancer. Cancer Cell. 2011; 19: 575–586. 10.1016/j.ccr.2011.04.008 21575859 PMC3142785

[24] Wang Y, Hayward S, Cao M, Thayer K, Cunha G Cell differentiation lineage in the prostate. Differentiation. 2001; 68: 270–279. 11776479 10.1046/j.1432-0436.2001.680414.x

[25] Thomson AA, Marker PC Branching morphogenesis in the prostate gland and seminal vesicles. Differentiation. 2006; 74: 382–392. 16916376 10.1111/j.1432-0436.2006.00101.x

[26] Donjacour AA, Cunha GR Assessment of prostatic protein secretion in tissue recombinants made of urogenital sinus mesenchyme and urothelium from normal or androgen-insensitive mice. Endocrinology. 1993; 132: 2342–2350. 7684975 10.1210/endo.132.6.7684975

[27] Goldstein AS, Lawson DA, Cheng D, Sun W, Garraway IP, Witte ON Trop2 identifies a subpopulation of murine and human prostate basal cells with stem cell characteristics. Proc Natl Acad Sci U S A. 2008; 105: 20882–20887. 10.1073/pnas.0811411106 19088204 PMC2603432

[28] Leong KG, Wang BE, Johnson L, Gao WQ Generation of a prostate from a single adult stem cell. Nature. 2008. 10.1038/nature07427 18946470

[29] Koltai T Clusterin: a key player in cancer chemoresistance and its inhibition. Onco Targets Ther. 2014; 7: 447–456. 10.2147/OTT.S58622 24672247 PMC3964162

[30] Higano CS Potential use of custirsen to treat prostate cancer. Onco Targets Ther. 2013; 6: 785–797. 10.2147/OTT.S33077 23836992 PMC3699352

[31] Stoyanova T, Goldstein AS, Cai H, Drake JM, Huang J, Witte ON Regulated proteolysis of Trop2 drives epithelial hyperplasia and stem cell self-renewal via beta-catenin signaling. Genes Dev. 2012; 26: 2271–2285. 10.1101/gad.196451.112 23070813 PMC3475800

[32] Trerotola M, Cantanelli P, Guerra E, Tripaldi R, Aloisi AL, Bonasera V, et al Upregulation of Trop-2 quantitatively stimulates human cancer growth. Oncogene. 2013; 32: 222–233. 10.1038/onc.2012.36 22349828

[33] Bhatia-Gaur R, Donjacour AA, Sciavolino PJ, Kim M, Desai N, Young P, et al Roles for Nkx3.1 in prostate development and cancer. Genes Dev. 1999; 13: 966–977. 10215624 10.1101/gad.13.8.966 PMC316645

[34] Kim MJ, Cardiff RD, Desai N, Banach-Petrosky WA, Parsons R, Shen MM, et al Cooperativity of Nkx3.1 and Pten loss of function in a mouse model of prostate carcinogenesis. Proc Natl Acad Sci U S A. 2002; 99: 2884–2889. 11854455 10.1073/pnas.042688999 PMC122442

[35] Van de Sande T, Roskams T, Lerut E, Joniau S, Van Poppel H, Verhoeven G, et al High-level expression of fatty acid synthase in human prostate cancer tissues is linked to activation and nuclear localization of Akt/PKB. J Pathol. 2005; 206: 214–219. 15880754 10.1002/path.1760

[36] Ghosh S, Lau H, Simons BW, Powell JD, Meyers DJ, De Marzo AM, et al PI3K/mTOR signaling regulates prostatic branching morphogenesis. Dev Biol. 2011; 360: 329–342. 10.1016/j.ydbio.2011.09.027 22015718 PMC3225010

[37] Yue S, Li J, Lee SY, Lee HJ, Shao T, Song B, et al Cholesteryl ester accumulation induced by PTEN loss and PI3K/AKT activation underlies human prostate cancer aggressiveness. Cell Metab. 2014; 19: 393–406. 10.1016/j.cmet.2014.01.019 24606897 PMC3969850

[38] Lei Q, Jiao J, Xin L, Chang CJ, Wang S, Gao J, et al NKX3.1 stabilizes p53, inhibits AKT activation, and blocks prostate cancer initiation caused by PTEN loss. Cancer Cell. 2006; 9: 367–378. 16697957 10.1016/j.ccr.2006.03.031

[39] Wang S, Garcia AJ, Wu M, Lawson DA, Witte ON, Wu H Pten deletion leads to the expansion of a prostatic stem/progenitor cell subpopulation and tumor initiation. Proc Natl Acad Sci U S A. 2006; 103: 1480–1485. 16432235 10.1073/pnas.0510652103 PMC1345717

[40] Choi N, Zhang B, Zhang L, Ittmann M, Xin L Adult murine prostate basal and luminal cells are self-sustained lineages that can both serve as targets for prostate cancer initiation. Cancer Cell. 2012; 21: 253–265. 10.1016/j.ccr.2012.01.005 22340597 PMC3285423

[41] Wang ZA, Mitrofanova A, Bergren SK, Abate-Shen C, Cardiff RD, Califano A, et al Lineage analysis of basal epithelial cells reveals their unexpected plasticity and supports a cell-of-origin model for prostate cancer heterogeneity. Nat Cell Biol. 2013; 15: 274–283. 10.1038/ncb2697 23434823 PMC3743266

[42] Wang X, Kruithof-de Julio M, Economides KD, Walker D, Yu H, Halili MV, et al A luminal epithelial stem cell that is a cell of origin for prostate cancer. Nature. 2009; 461: 495–500. 10.1038/nature08361 19741607 PMC2800362

