# Supplementary figures and images for: Pten Regulates Epithelial Cytodifferentiation during Prostate Development
Source: PLoS One. 2015 Jun 15;10(6):e0129470. doi: 10.1371/journal.pone.0129470 (PMC4468205; doi:10.1371/journal.pone.0129470)

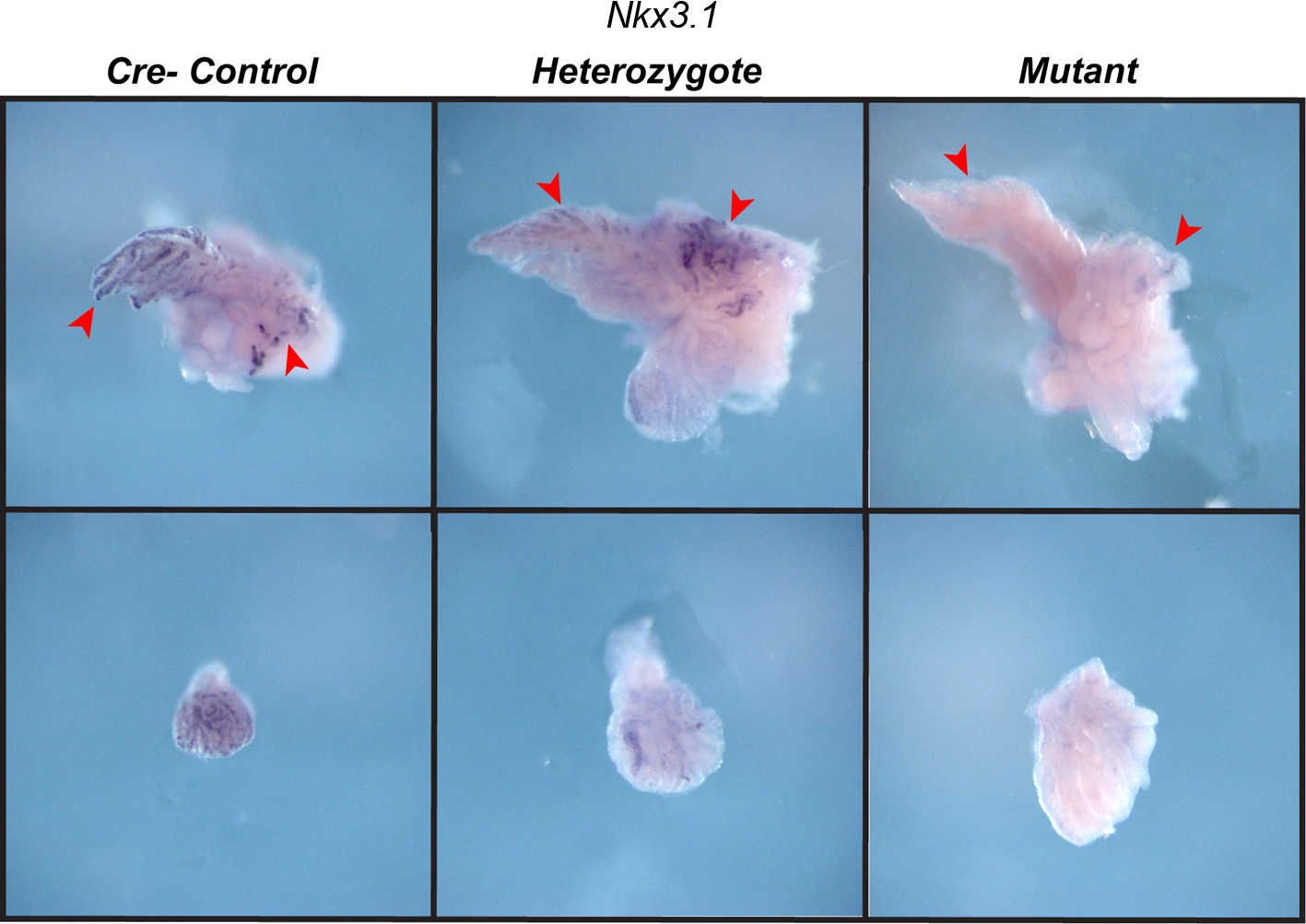

Supplement: S1 Fig — Cre- controls and Pten heterozygotes (Nkx3.1:Cre;Pten Fl/+) both express Nkx3.1 in all lobes. The anterior prostate (AP), dorsal lateral prostate (DL) and urethra (UR) are indicated. Red arrows indicate presence of stain in the controls and absence in the mutant. (TIF) [file pone.0129470.s001.tif]

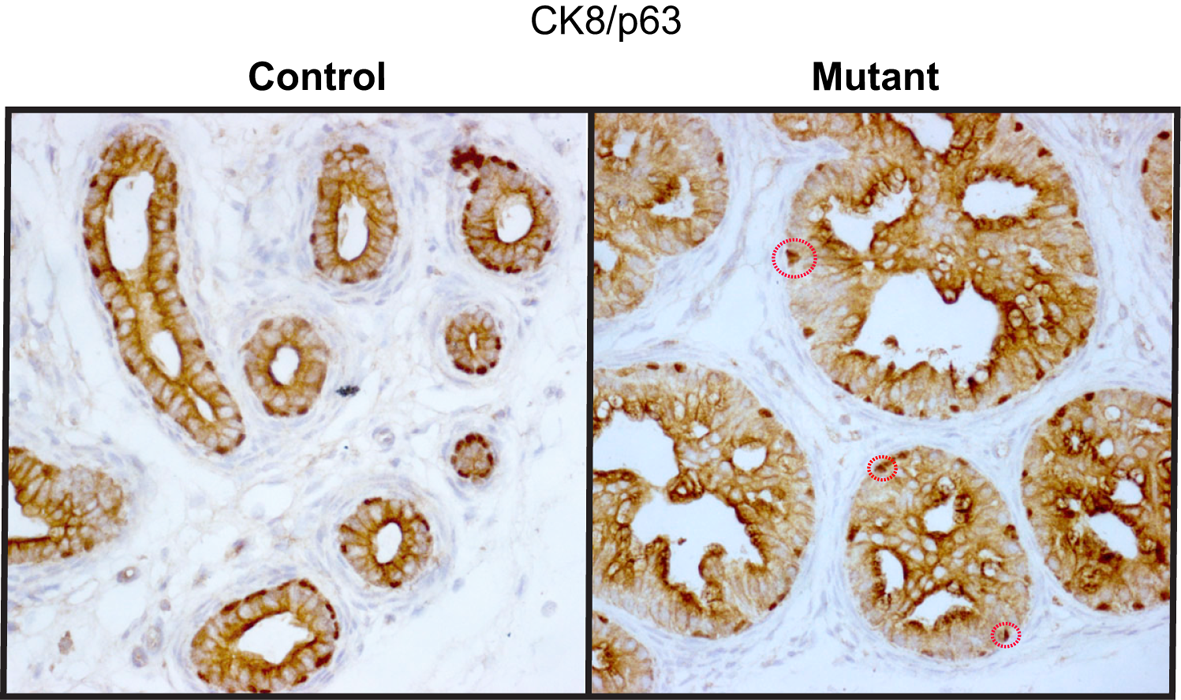

Supplement: S2 Fig — Mutant ducts are filled with luminal cells expressing high levels of cytoplasmic CK8. Red circles highlight basal cells that express nuclear p63. (TIF) [file pone.0129470.s002.tif]
